# Supplementary material for: Manipulating azobenzene photoisomerization through strong light–molecule coupling
Source: Nat Commun. 2018 Nov 8;9:4688. doi: 10.1038/s41467-018-06971-y (PMC6224570; doi:10.1038/s41467-018-06971-y)
Supplement: Supplementary file 1 — Supplementary Information [file 41467_2018_6971_MOESM1_ESM.pdf]

Supplementary Information:

Manipulating azobenzene photoisomerization through strong light-molecule coupling

J. Fregoni *et al.*[*]

Supplementary Note 1: Transition dipole moment and polaritonic energy splitting

As mentioned in the main text, the interaction between light and molecule strictly depends on the transition dipole moment component along the polarization of the electromagnetic mode, for each nuclear coordinate. Therefore, the polaritonic energy splitting dependence on the nuclear coordinates is embodied into the dependence on the transition dipole moment:

$$\hat{H}_{\mathrm{int}}(Q) = g\hat{\boldsymbol{\mu}}_{\mathbf{S_0},\mathbf{S_1}}(Q) \cdot \boldsymbol{\lambda} \left(\hat{b}^{\dagger} + \hat{b}\right). \tag{1}$$

The transition dipole components between the ground and first excited state for the isolated azobenzene molecule are shown in Supplementary Figure 1. It is evident that the range of variation of the transition dipole moment on the geometry can cover several Debyes, depending on the alignment of the molecule. In this work, we take the field as polarized along z, i.e. the axis perpendicular to the molecular plane: as a consequence, along the avoided crossing line, the transition dipole z-component varies from zero to few Debyes. The splitting magnitude and profile also depends strictly on the field orientation with respect to the molecule. Such conditions gives origin to splittings ranging from zero to about 100 meV for a coupling constant g of 0.010 au.

In Supplementary Figure 2 two strong coupling avoided crossing profiles with g of 0.010 au, the field polarized along the z axis and two different photon energies are compared. In the plot, such splitting is defined as the contribution to the energy splitting between the upper and lower polariton due to the strong-coupling matrix element. The profile presented in the Panel a) corresponds to the case where the $|\mathrm{S}_0, 1\rangle$ energy is set to E_{ph} 1.3 eV, while the Panel b) is the E_{ph} 2.2 eV case. The splitting is evaluated analytically for a two level system composed of the states $|\mathrm{S}_0, 1\rangle$ and $|\mathrm{S}_1, 0\rangle$, taking into account the dependence of the dipoles on the nuclear coordinates:

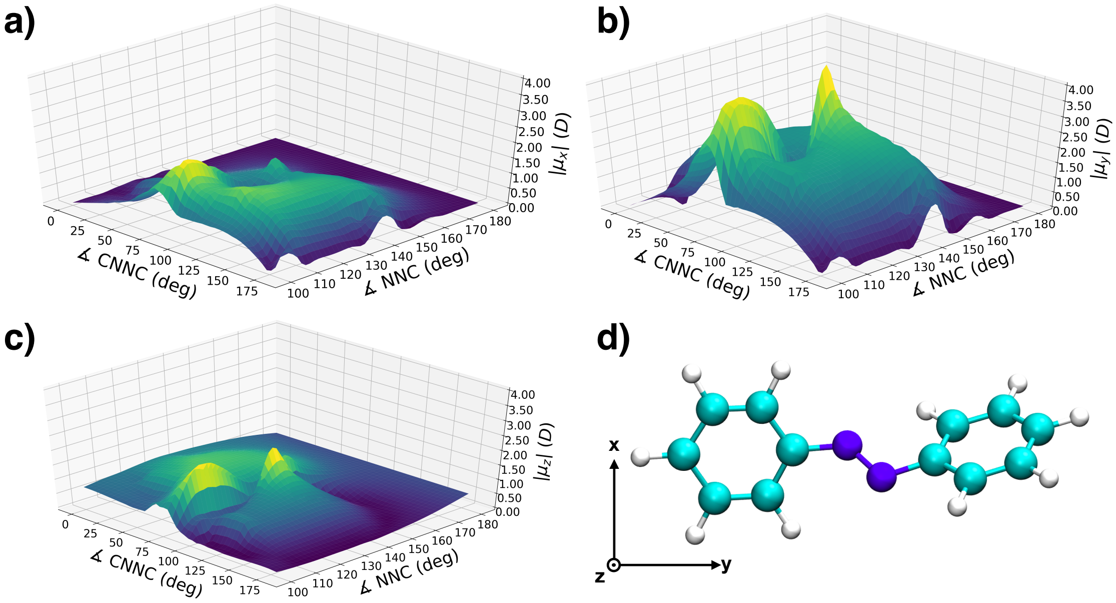

Supplementary Figure 1: **Transition dipole moment in azobenzene. a), b), c)** Transition dipole moments components along the x, y and z direction respectively, as a function of the $CNNC$ and NNC coordinates. **d)** The orientation of the trans azobenzene with respect to the x, y and z.

$$\Delta E^{\mathrm{sc}}_{+,-} = -\left|H_{\mathrm{S}_1,0} - H_{\mathrm{S}_0,1}\right| + \sqrt{\left|H_{\mathrm{S}_0,1} - H_{\mathrm{S}_1,0}\right|^2 + 4g^2\mu_{\mathrm{z}}^2}. \quad (2)$$

As highlited in the main text, the position of the contribution of the interaction to the polaritonic splitting can be tuned by changing the photon frequencies E_{ph} (1.3 eV and 2.2 eV respectively). For both the analyzed cases the splitting goes to zero when approaching the $CNNC$ value of 180°, signature of an intersection line between the uncoupled states with a zero interaction element. The magnitude of such contribution, instead, shows dramatic and non trivial dependence on the nuclear coordinates in both cases. As the azobenzene molecule moves out of the plane through torsion of the $CNNC$ dihedral angle, the splitting value increases drastically, carrying a non-trivial dependence on the nuclear coordinates.

As shown in Supplementary Figure 2 the Rabi splitting approaches the zero as the $CNNC$ angle moves towards 180 (planar molecule). The zero transition dipole moment perpendicular to the molecule together with the field polarization along the same component (used in the

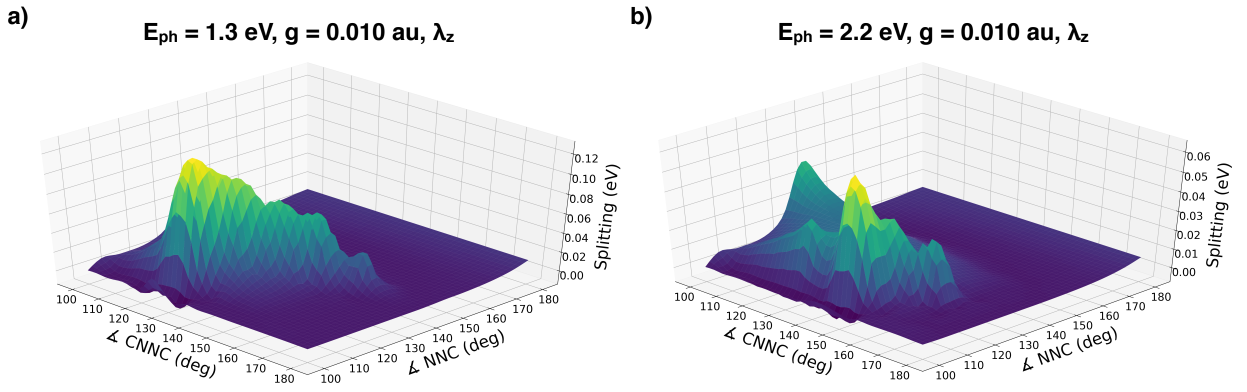

Supplementary Figure 2: **Analytical Rabi Splitting for a model two-states azobenzene. Panel a)** Splitting profiles for the E_{ph} 1.3 eV **Panel b)** and 2.2 eV case.

current work) suggests the presence of a light-induced conical intersection. In Supplementary Figure 3 the polaritonic conical intersections for the 1.3 eV and the 2.2 eV cases are shown. As anticipated in the main text, the position of the polaritonic conical intersection can be tuned by changing the photon frequency. For the 1.3 eV case, the intersection is located at 180 along the $CNNC$ coordinate and 155.33 along the NNC (Supplementary Figure 3a,b); for the 2.2 eV case, the intersection is located at 180 along the $CNNC$ coordinate and 135 along NNC (Supplementary Figure 3c,d).

Together with the different position of the polaritonic conical intersection, the different crossing geometry entails a substantially different shape of the polaritonic potential energy surfaces in the surroundings of the crossing seam. The effects of such difference in the two studied cases are discussed in the main text and in the next section (Supplementary Figures 3, 4).

Supplementary Note 2: Strong coupling non-adiabatic dynamics

In this section we overview the conditions and the supplementary characterization for the two performed dynamics simulations. The sampling was performed by running 10 ps of equilibration dynamics on the ground state of azobenzene, absent field and with a single trajectory. The initial velocities for such trajectory are distributed according to a Maxwell-Boltzmann distribution at room temperature, and the sampling dynamics is performed on the ground state

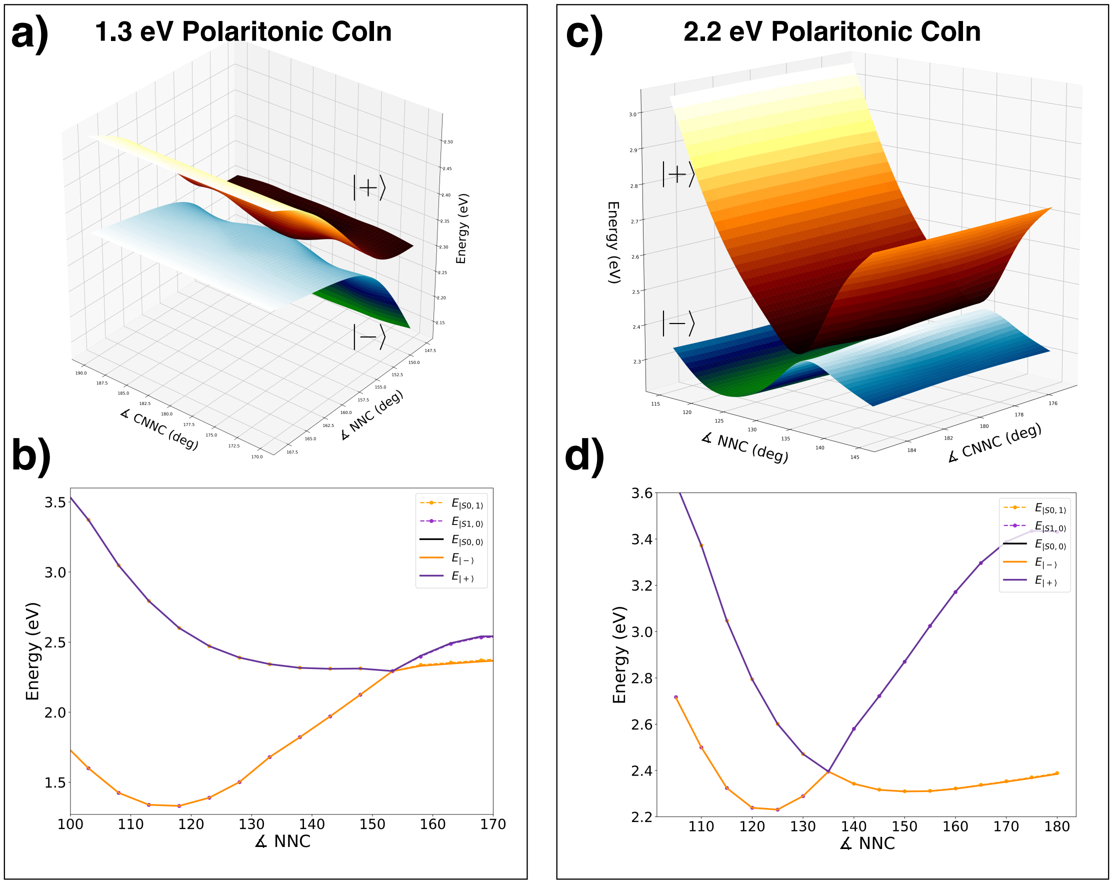

Supplementary Figure 3: **Polaritonic conical intersection and section of the polaritonic potential energy surfaces. a), b)** the 1.3 eV case **c), d)** the 2.2 eV case. The blue surfaces are the lower polaritons, the orange surfaces are the upper polaritons. The color scheme for the potential energy curves is mantained from the main text (Figures 2, 4 and 5)

with a Bussi-Parrinello stochastic thermostat, with a time constant of 10 fs. We sample 300 sets of positions and velocities from the equilibration dynamics. We retain the same sampling conditions for all the following dynamics in order to highlight the differences in the mechanism driven by different coupling conditions. In this way, we are able to ascribe the differences in the evolution of the wavepackets to the difference in the polaritonic potential energy surfaces. For each sampled trajectory, we compute the polaritonic states and we excite it through a Franck-Condon excitation to the upper polaritonic state. Each trajectory runs and independent dynamics for 2 ps, with a time step of 0.1 fs. We refer to Figure 4 of the main text for the discussion about the mechanism change induced by the strong coupling regime. In Supplementary Figures 4a and Supplementary Figure 4b we propose a scheme for the splitting of the

wavepacket along the oscillation coordinates (Figure 4a and 4d of the main text) for the E_{ph} 1.3 eV and 2.2 eV respectively.

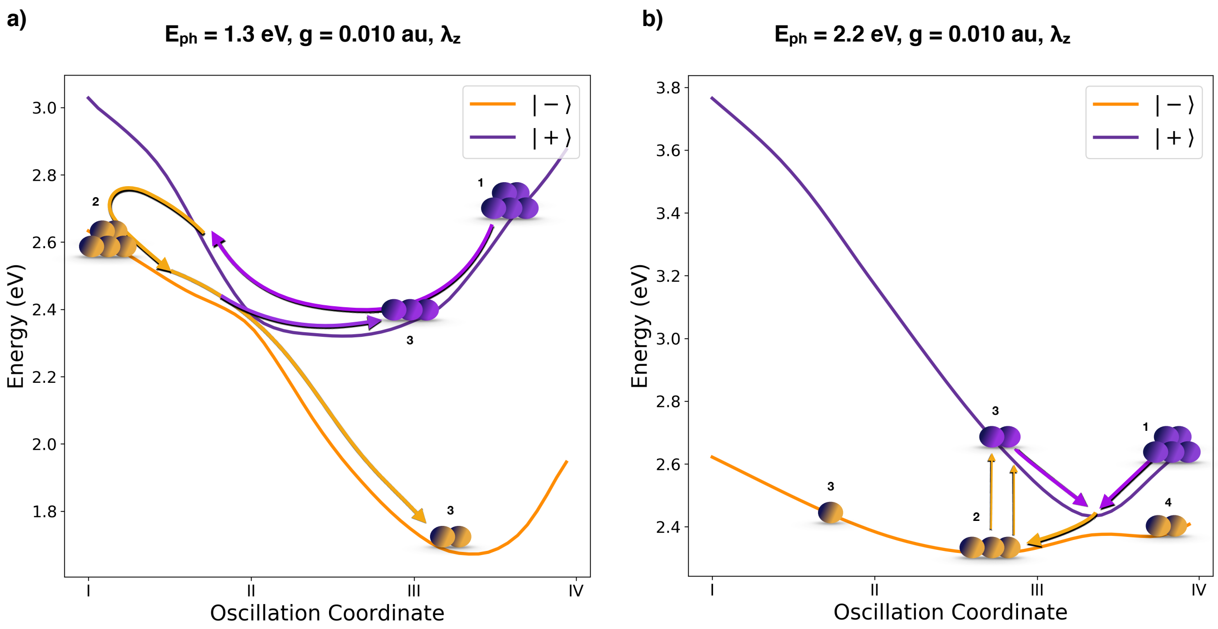

Supplementary Figure 4: **Scheme of the different wavepacket splitting.** The splitting is driven by the strong coupling avoided crossing shape in the E_{ph} **a)**1.3 eV and **b)** 2.2 eV respectively. The color scheme is mantained from the main text (Figures 2, 4 and 5)

The complete movies of the dynamics are presented as standalone files of the supplementary material. Interpreting together the hereby scheme and the proposed movies, it becomes evident how the different shaping of the avoided crossing due to different coupling conditions impacts the dynamics. In the characterization of the dynamics, we also analyzed the average oscillation of the $CNNC$ and NNC angles, together with the oscillation of the energy difference between the polaritonic states (Supplementary Figure 5 and Supplementary Figure 6). The oscillation of the angles (Supplementary Figure 5) in the first 500 fs of the dynamics allowed us to extract the range of the average oscillation coordinate for the wavepacket in the two cases. The oscillation of energy in Supplementary Figure 6 is a signature of the hopping between polaritonic states. In particular, the first pronounced peak implies that all the trajectories move toward the minimum of the upper polariton before splitting. As the trajectories split close to the strong-coupling avoided crossing, the oscillation in the energy difference damps

progressively, following the oscillation trend of populations presented in Figure 4c and 4f (main text).

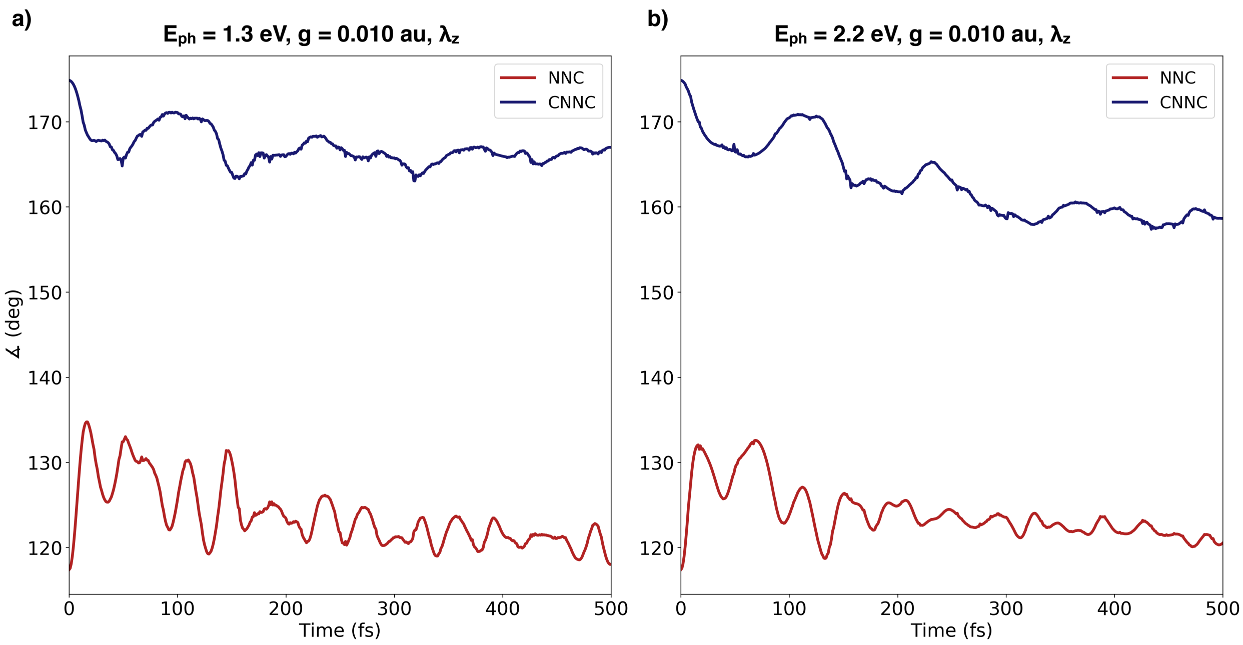

Supplementary Figure 5: **Oscillation of the** $CNNC$ **and** NNC **coordinates. a)** E_{ph} 1.3 eV **b)** 2.2 eV case, respectively.

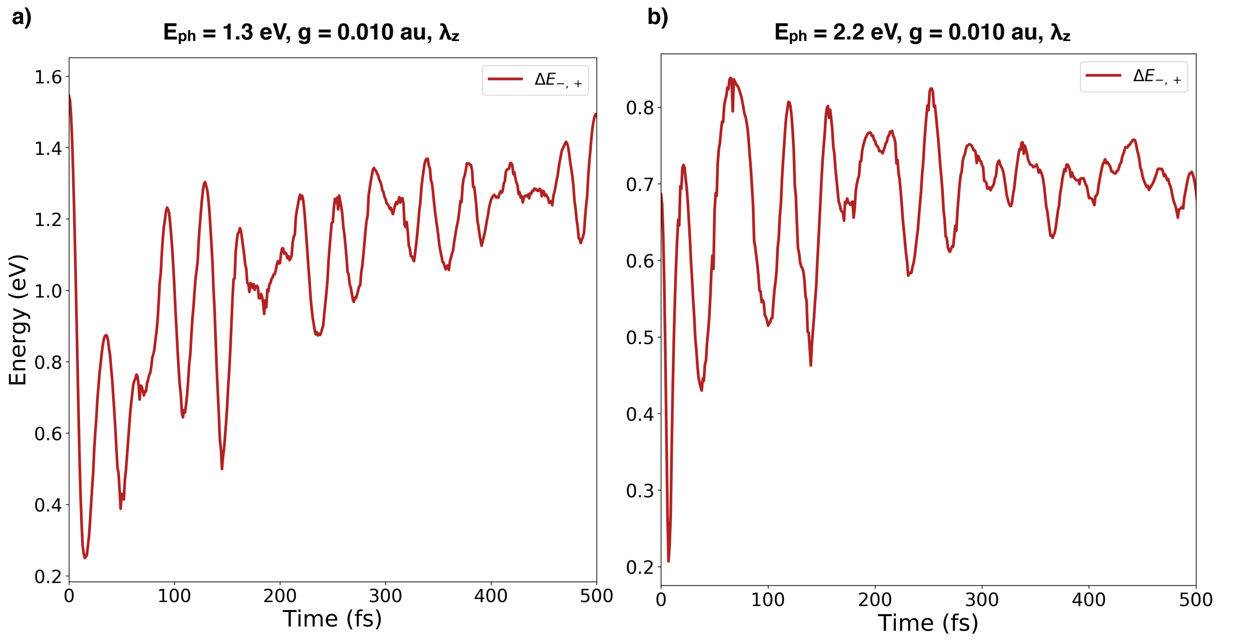

Supplementary Figure 6: **Oscillation of the energy difference between the** $|+\rangle$ **and** $|-\rangle$ **states. a)** E_{ph} 1.3 eV and **b)** 2.2 eV case, respectively.

In Supplementary Figure 7 we report the quantum yields dependence on the coupling constant g and the photon frequency. Such non-monotonic dependence can be approximately split in three domains: low frequency (less than 0.8 eV), middle frequency (0.8-2.0 eV) and high frequency (higher than 2.0 eV). In the middle frequency domain the yields are very low, $i.e.$, the photoisomerization is quenched. The origin of this behavior can be traced to the trapping in the lower polariton state, as discussed above. Remarkably, for g=0.005 au in the high frequency domain, we observe an increase of the yield, although very limited (but with statistical significance, the error being smaller than 1$\%$). The reason for such behavior is that the PPES are modified in such a way that the system approaches the electronic conical intersection with larger velocities. As a consequence, the trajectories perform hops with a slightly higher probability towards the cis branch.

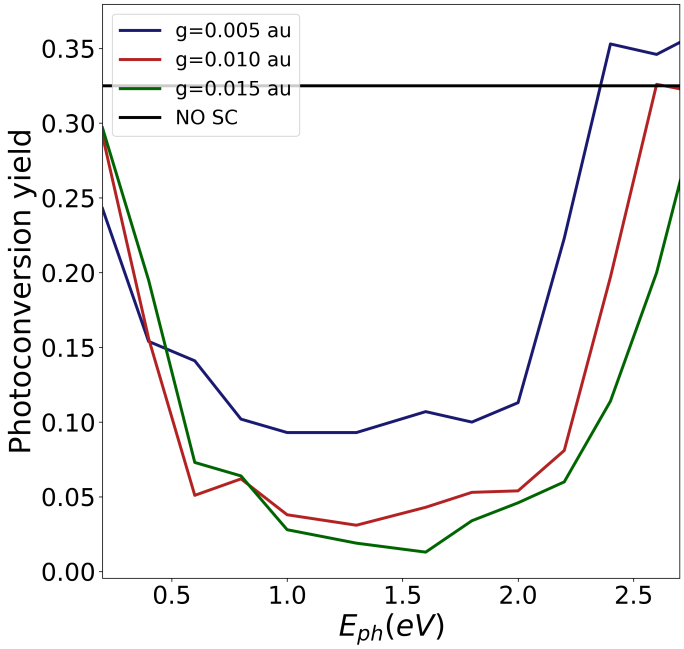

Supplementary Figure 7: **Dependence of the quantum yields on the photon energies and coupling constants.** The quenching of the reaction is dominated by the trapping mechanism for energies between 0.8 and 2.0 eV. For different energies, the quenching-by-trapping mechanism is suppressed: at low energies, for concomitance of the strong coupling avoided crossing and electronic conical intersection; at high energies, the trapping minimum becomes less deep and the wavepacket proceeds towards the conical intersection.

Finally, in the lower frequency domain we observe quantum yields that are recovering

towards the weak-coupling value. In this case, the polaritonic avoided crossing is close to the electronic conical intersection. Accordingly, the hopping of the trajectories to the lower polaritonic state and the reaching of the conical intersection become concurrent, leading back to a mechanism similar to that in the weak coupling regime and thus to similar yields. However, such low photon frequency becomes resonant with vibrational transitions, calling for a quantum mechanical description of the nuclear motion.

Supplementary Note 3: Cavity Losses

The results for the inclusion of the cavity losses is already discussed in the main text for the 1.3 eV case. Here we present the effect of explicitely including the cavity lifetime for the 2.2 eV case (Supplementary Figure 8). As for the 1.3 eV case, we take into account 4 cavity lifetimes: 10 fs, 50 fs, 100 fs (Supplementary Figures 8a,b,c respectively) and 150 fs.

As discussed in the main text, the inclusion of the cavity lifetime impacts the decay mechanism of the system: the driving process for the de-excitation of a polaritonic system becomes the loss of coherence between the polaritonic states (Supplmentary Figure 8d). As the 2.2 eV case is characterized by a quick population drop on the lower polariton, the overall relaxation mechanism is ruled by the lower polaritonic decay. Indeed, in the 2.2 eV case the whole dynamics occurs in a region where the $|\mathrm{S1}, 0\rangle$ and $|\mathrm{S0}, 1\rangle$ states are more mixed than in the 1.3 eV case: while in the 1.3 dynamics the evolution of the uncoupled and polaritonic states differs only at the oscillations peaks, where the wavepacket goes through the narrow crossing seam, in the 2.2 eV case (when no losses are considered) the populations of coupled and uncoupled states follow opposite trends. As shown in Figure 4f of the main text, when the lower polariton is completely populated (100 to 200 fs), the uncoupled populations are both about 0.5. The cavity loss, therefore, affects greatly the decay of both polaritonic states (Supplementary Figure c), in contrast with the 1.3 eV case, where the most affected state is the lower polariton. In particular, as the cavity loss becomes quicker, the polaritons tend to evolve as the uncoupled states earlier (Supplementary Figures 8 a,b,c) due to the loss of mixing. This behaviour be-

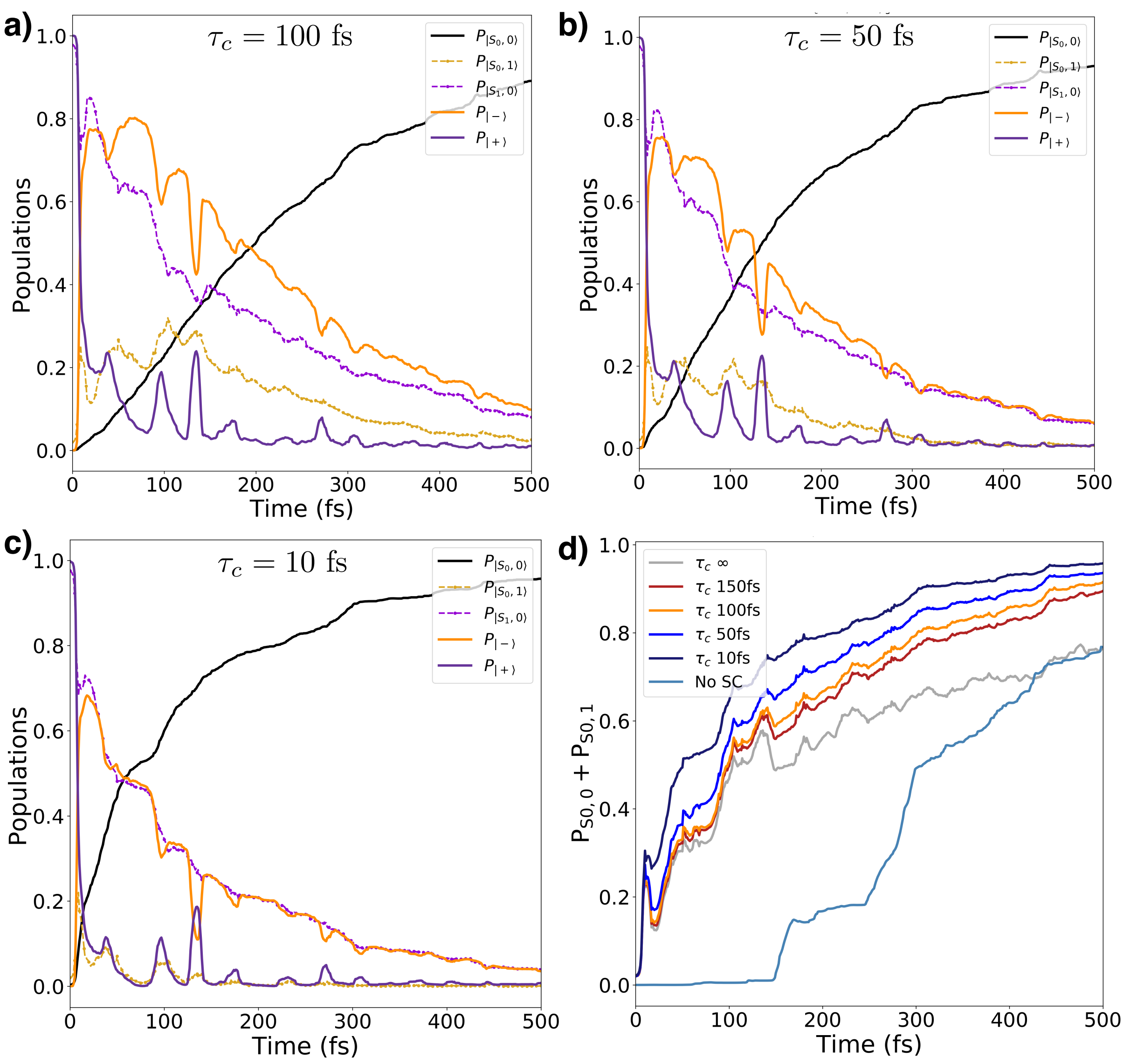

Supplementary Figure 8: **Cavity lifetimes for the 2.2 eV case.** Population evolution of the system with the inclusion of the cavity losses. The cavity lifetimes are: **a)** 100 fs, **b)** 50 fs, **c)** 10 fs. **d)** Ground state population retrieval for the 2.2 eV dynamics. The color scheme is mantained from the main text (Figures 2, 4 and 5)

comes evident in Supplementary Figure 8d, where the major differences between the ground state population retrieval are observed between the $\tau_c = \infty$ case and the $\tau_c = 150\ fs$ case. As addressed in the main text, we can conclude that if the wavepacket evolves in a region where the polaritonic states are mixed in nature, the decoherence between light and matter becomes the main decay channel for such system.

Supplementary Note 4: Gauge choice and extendend Jaynes-Cummings model

We hereby present the plots of polaritonic states obtained numerically starting from the bare electronic states as reported in the electronic structure paragraph of the Methods section. In this numerical polaritonic state calculation, we compared compare the dipolar and extended Jaynes-Cummings Hamiltonians. The dipolar Hamiltonian is obtained through the Göppert-Meyer gauge transformation starting from a minimal coupling Hamiltonian in the Coulomb gauge (C. Cohen-Tannoudj, *Photons and atoms*, Wiley Interscience, 1997). Both the hamiltonians are composed by the molecular and photonic contributions and the light-matter interaction term:

$$\hat{H} = \hat{H}_{\rm mol} + \hat{H}_{\rm ph} + \hat{H}_{\rm int}. \tag{3}$$

The light-matter interaction term in the dipolar formulation is:

$$\hat{H}_{\rm dip} = i\hat{\boldsymbol{\mu}} \cdot \boldsymbol{\lambda}\sqrt{\omega_{\rm ph}}A_0 \left(\hat{b} - \hat{b}^\dagger\right) + 2\left(\mu \cdot \lambda\right)^2 A_0^2, \tag{4}$$

where A_0 is the vector potential intensity, $\boldsymbol{\lambda}$ is the polarization of the field, $\omega_{\rm ph}$ is the frequency of the resonant mode, $\hat{b}$ and $\hat{b}^\dagger$ are the bosonic annihilation and creation operators respecitvely and $\hat{\boldsymbol{\mu}}$ is the dipolar operator. We distinguish two interaction orders in the vector potential. The first order terms embody the interaction between states differing for one in the photon number:

$$iA_0\sqrt{\omega_{\rm ph}}\langle {\rm n}, {\rm p}|\hat{\mu}\left(\hat{b} - \hat{b}^\dagger\right)|{\rm n}', {\rm p} \pm 1\rangle = \pm iA_0\sqrt{\omega_{\rm ph}}\sqrt{p+1}\mu_{\rm n,n'} \tag{5}$$

where $|{\rm n}, {\rm p}\rangle$ is the basis of the uncoupled states (see Methods section of the main text). The first order terms consist of two kind of interactions: one mixing states with the same electronic index,

$$iA_0\sqrt{\omega_{\rm ph}}\langle {\rm n}, {\rm p}|\hat{\mu}\left(\hat{b} - \hat{b}^\dagger\right)|{\rm n}, {\rm p} \pm 1\rangle = \pm iA_0\sqrt{\omega_{\rm ph}}\sqrt{p+1}\mu_{\rm n,n}, \tag{6}$$

and the other depening on the transition dipole moments, which is characteristic of the strong coupling regime,

$$iA_0\sqrt{\omega_{\rm ph}}\langle {\rm n},{\rm p}|\hat{\mu}\left(\hat{b}-\hat{b}^{\dagger}\right)|{\rm n}',{\rm p}\pm 1\rangle = \pm iA_0\sqrt{\omega_{\rm ph}}\sqrt{p+1}\mu_{{\rm n}',{\rm n}}, \tag{7}$$

with $n \neq n'$. To compute the second order terms, we exploit a resolution of identity:

$$A_0^2\langle {\rm n},{\rm p}|\hat{\mu}^2|{\rm n}',{\rm p}'\rangle = A_0^2\sum_{i}^{N_{\rm st}}\langle {\rm n},{\rm p}|\hat{\mu}|{\rm i},{\rm p}'\rangle\langle {\rm i},{\rm p}|\hat{\mu}|{\rm n}',{\rm p}'\rangle\delta_{{\rm p},{\rm p}'}. \tag{8}$$

Due to the absence of any bosonic operator in the second order interaction terms, these kind of terms act only on the electronic part. Therefore, a shift of the diagonal elements of the Hamiltonian is provided by the terms:

$$\langle {\rm n},{\rm p}|\hat{\mu}^2|{\rm n},{\rm p}\rangle = A_0^2\sum_{i=1}^{N_{\rm st}}\langle {\rm n},{\rm p}|\hat{\mu}|{\rm i},{\rm p}\rangle\langle {\rm i},{\rm p}|\hat{\mu}|{\rm n},{\rm p}\rangle, \tag{9}$$

together with a correction to the isolated molecule interaction between electronic states,

$$\langle {\rm n},{\rm p}|\hat{\mu}^2|{\rm n}',{\rm p}\rangle = A_0^2\sum_{i=1}^{N_{\rm st}}\langle {\rm n},{\rm p}|\hat{\mu}|{\rm i},{\rm p}\rangle\langle {\rm i},{\rm p}|\hat{\mu}|{\rm n}',{\rm p}\rangle, \tag{10}$$

with $n \neq n'$. The extended Jaynes-Cummings model disregards the permanent dipole contributions and the second order terms and it can be recast in a dipolar-like shape:

$$\hat{H} = \hat{H}_{\rm mol} + \hat{H}_{\rm ph} + \hat{\boldsymbol{\mu}}_{\bf tr}\cdot\boldsymbol{\lambda}\sqrt{\omega_{\rm ph}}A_0\left(\hat{b}+\hat{b}^{\dagger}\right) \tag{11}$$

where $\hat{\boldsymbol{\mu}}_{\bf tr}$ is the transition dipole moment operator acting on the electronic states and the ratio $g/\omega_{\rm ph}$ is $A_0/\sqrt{\omega_{\rm ph}}$. The problem of the gauge choice in strongly coupled light-matter systems has been presented by Flick[1] *et. al.* In particular, they emphasized that for high $g/\omega_{\rm ph}$ ratios (greater than 0.4 au) the full dipolar light-matter Hamiltonian must be considered. Such $g/\omega_{\rm ph}$ ratio is peculiar of vibro-polaritonic (photon resonant with vibrational transitions) cou-

pled systems, which are characterized by low frequencies together with high fields. Under such conditions, the second order terms are non negligible as they impact deeply the bond lengths. Nevertheless, within the conditions where the electronic transitions are resonant with a cavity mode, the involved g/ω_{ph} ratios set the system in a regime where higher order terms can be disregarded (ranging from 0.20 au to 0.12 au for the E_{ph} 1.3 eV and 2.2 eV respectively). For the sake of completeness, we numerically verified the stability of our approximation for the conditions used in this work. We computed the states involved in the non-adiabatic dynamics by taking into account two electronic states ($|\mathrm{n}\rangle$=$|\mathrm{S}_0\rangle$,$|\mathrm{S}_1\rangle$) and two occupation numbers for the photon ($|\mathrm{p}\rangle$=$|0\rangle$,$|1\rangle$). The results for the 1.3 eV and 2.2 eV cases are reported in Supplementary Figure 9, where it is shown that the additional terms appearing in the dipolar formulation can be disregarded within our system conditions. Even though negligible, the minor differences between the polaritonic states within the two models shown in Supplementary Figure 9 (full lines) are amenable to the truncation of the occupation number space. When considering the truncated space, we disregard the interaction terms of Supplementary Equation 6. In particular, the state $|\mathrm{S}_0, 1\rangle$ (and $|\mathrm{S}_1, 1\rangle$, respectively) do not interact with $|\mathrm{S}_0, 2\rangle$ ($|\mathrm{S}_1, 2\rangle$) in the truncated space, due to the absence of the $|2\rangle$ occupation number. In order to reach the agreement between the two models, we include in the space one more occupation state number ($|2\rangle$) for each electronic state. As shown in Supplementary Figure 10, the two models converge to the same results despite the difference in the uncoupled states (dotted line). Such difference in the uncoupled states is due to the diagonal shift coming from the second order term of the dipolar Hamiltonian (Supplementary Equation 9).

On the basis of the negligible differences outlined with the numerical results presented in this section, we decided to rely on the extended Jaynes-Cummings Hamiltonian for our specific conditions. Nevertheless, it is to emphasize once more that the range of stability of this approximation must be carefully checked when treating strongly coupled systems as addressed by Flick *et. al*(J. Flick, M. Ruggenthaler, H. Appel, A. Rubio, *Proc. Natl. Acad. Sci. USA*, 114(12), 3026, 2017).

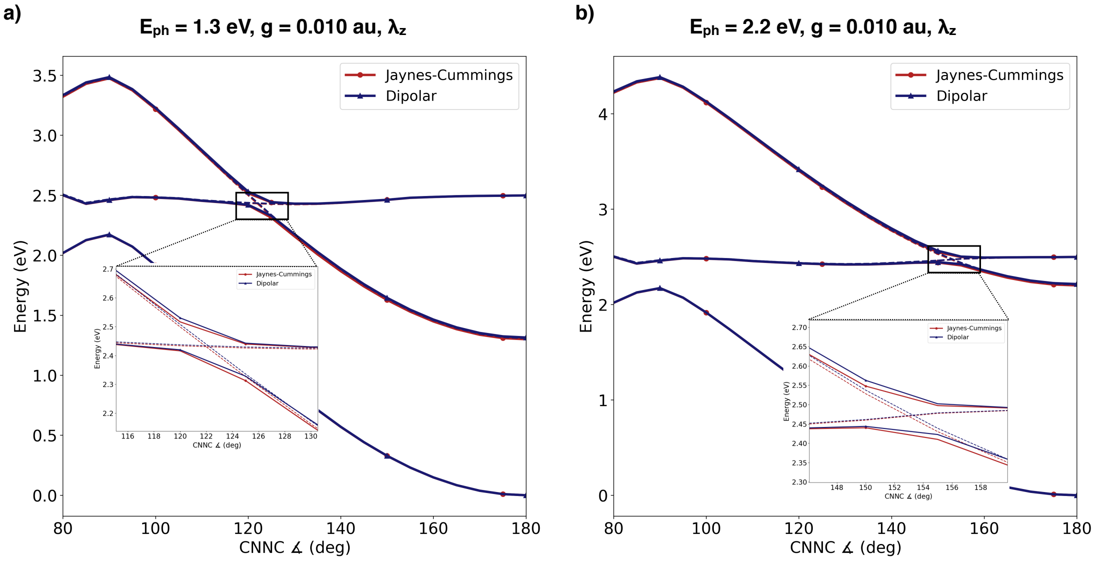

Supplementary Figure 9: **Numerical comparison of the light-matter interaction Hamiltonians. a)** E_{ph} 1.3 eV and **b)** 2.2 eV case respectively in the truncated space with $|0\rangle$ and $|1\rangle$ photon occupation state numbers. The coupling constant is g 0.010 au and the field is oriented along the z axis of the molecule. The polaritonic states are computed with the Jaynes-Cummings model (dark red full lines) and dipolar model (dark blue full lines) starting from the basis of the uncoupled states (dashed lines).

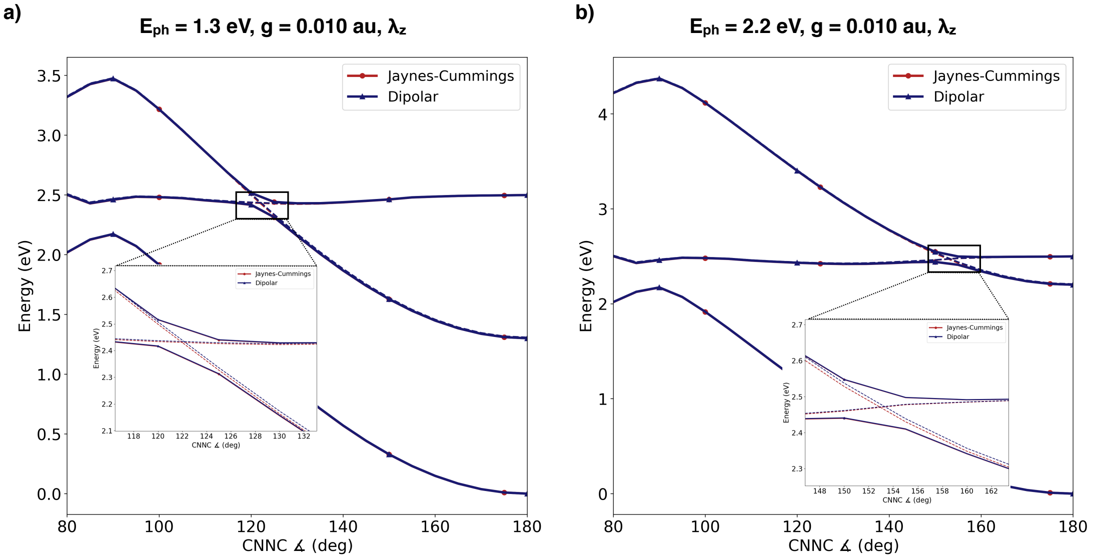

Supplementary Figure 10: **Numerical comparison of the light-matter interaction Hamiltonians. a)** E_{ph} 1.3 eV and **b)** 2.2 eV case respectively. In this calculation, 6 states are included: the space is spanned by the direct product of the electronic states times the occupation numbers for the photon. The colorscheme is the same as in Supplementary Figure 9

Supplementary Methods

The following notation is used in the flowchart:

1. $|\mathrm{K}\rangle$ and U_{K} are electronic states and energies (approximate eigenfunction of the electronic Hamiltonian for the free molecule). In the present case we only presenteed $K = S_0, S_1$. The polaritonic "diabatic" states are obtained as products $|\mathrm{K}, \mathrm{n}\rangle$ (n being the photon occupation number), as mentioned in the main text.

2. $\boldsymbol{\mu}_{\mathrm{KL}} = \langle \mathrm{K} \,|\hat{\mu}|\, \mathrm{L} \rangle$ are the matrix elements of the molecular dipole.

3. $|\mathrm{A}\rangle$ and E_{A} are the polaritonic adiabatic states (linear combinations of $|\mathrm{K}, \mathrm{n}\rangle$) and energies.

4. The polaritonic wavefunction is $\Psi(t) = \sum_{\mathrm{A}} C_{\mathrm{A}}(t)|\mathrm{A}\rangle$

5. The state where the trajectory is running on is the "current" adiabatic state (labelled Γ).

6. $T_{\Gamma \rightarrow \mathrm{A}}$ is the surface hopping transition probability.

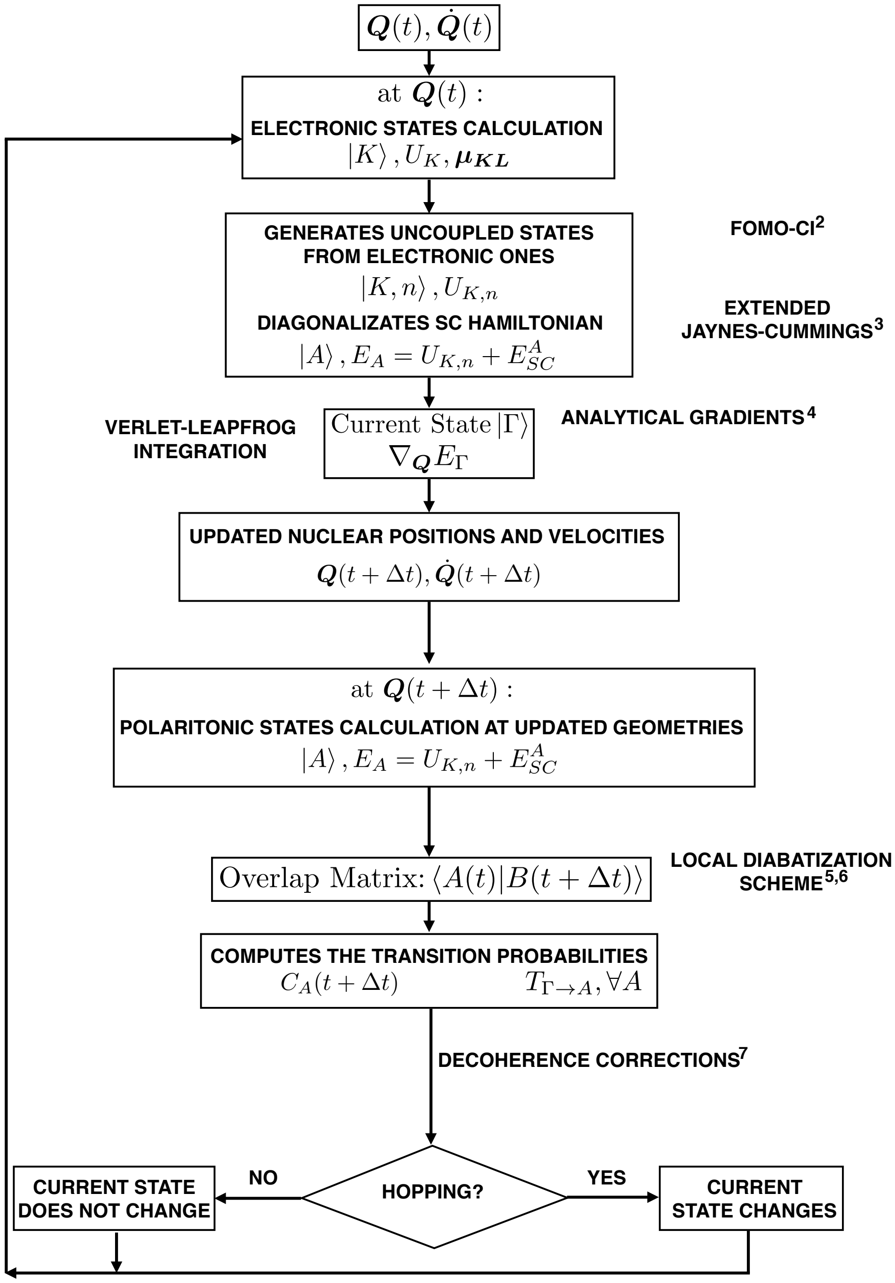

Supplementary Figure 11: **Flow chart for the algorithm applied to propagate each trajectory.** For each sampled trajectory (see Main Text) a Franck-Condon excitation is performed to the upper polariton. At each time step the electronic and polaritonic energies and coeffiecients are computed. The gradient of the polaritonic energy is calculated to propagate the classical nuclear tajectories. The overlap between the polaritonic wavefunction at the begin and the end of the timestep is computed to evaluate the transition probability, accordingly to the local diabatization scheme on which we rely on. The decoherence correction is then applied and, if a hopping has occurred, the nuclear kinetic energy of the trajectory is readjusted to ensure total energy conservation. The propagation is then resumed. The statistical treatment of all the trajectories retrieves the branching of the wavepacket on the potential energy surfaces.

Supplementary References

(1) Flick, J., Ruggenthaler, M., Appel, H. & Rubio, A Atoms and molecules in cavities, from weak to strong coupling in quantum-electrodynamics (QED) chemistry. *Proc. Natl. Acad. Sci. USA* **114**, 3026-3034 (2017).

(2) Cusati, T., Granucci, G., Martinez-Nunez, E., Martini, F., Persico, M. & VÃązquez, S. Semiempirical Hamiltonian for Simulation of Azobenzene Photochemistry. *J. Phys. Chem. A* **116**, 98-110 (2011).

(3) Galego, J., Garcia-Vidal, F. J. & Feist, J. Cavity-Induced Modifications of Molecular Structure in the Strong-Coupling Regime. *Phys. Rev. X* **5**, 041022 (2015).

(4) Granucci, G. & Persico, M. Gradients for configuration interaction energies with spinâĂŘorbit coupling in a semiempirical framework. *J. Comput. Chem.* **32**, 2690 (2011).

(5) Granucci, G., Persico, M. & Toniolo, A. Surface hopping trajectory simulations with spin-orbit and dynamical couplings. *J. Chem. Phys.* **114**, 10608 (2001).

(6) Plasser, F., Granucci, G., Pittner, J., Barbatti, M., Persico, M. & Lischka, H. Surface hopping dynamics using a locally diabatic formalism: Charge transfer in the ethylene dimer cation and excited state dynamics in the 2-pyridone dimer, *J. Chem. Phys.* **137**, 22A514(2012).

(7) Granucci, G., Persico, M. & Zoccante, A. Surface hopping trajectory simulations with spin-orbit and dynamical couplings. *J. Chem. Phys.* **133**, 134111 (2010).
