# Supplementary material for: Manipulating azobenzene photoisomerization through strong light–molecule coupling
Source: Nat Commun. 2018 Nov 8;9:4688. doi: 10.1038/s41467-018-06971-y (PMC6224570; doi:10.1038/s41467-018-06971-y)
Supplement: Supplementary file 2 — Description of Additional Supplementary Files [file 41467_2018_6971_MOESM2_ESM.docx]

Supplementary Movies 1 and 2: the upper panel is the upper polaritonic potential energy surface, the middle panel is the middle polaritonic potential energy surface and the lower panel is the ground state. Each dot represents a trajectory in the conformational space: the light blue ones are referred to the upper polariton, orange ones to the lower polaritons and black to the ground state.

Supplementary Movie 3: the upper panel is the first electronic excited state of the isolated azobenzene molecule, the lower panel is the ground state. Orange dots are the trajectories on the first excited state and the black dots are the trajectories on the ground state.
